# Older adult’s experience of chronic low back pain and its implications on their daily life: Study protocol of a systematic review of qualitative research

**DOI:** 10.1186/s13643-018-0742-5

**Published:** 2018-05-24

**Authors:** Arnold Y. L. Wong, Katarina Sjögren Forss, Jenny Jakobsson, Veronika Schoeb, Christine Kumlien, Gunilla Borglin

**Affiliations:** 1 Department of Rehabilitation Sciences, Faculty of Health and Social Sciences, The Hong Kong Polytechnic University, Hung Hom, Hong Kong, Special Administrative Region of China; 2 Department of Care Science, Faculty of Health and Society, Malmö University, SE-205 06 Malmö, Sweden; 3 Department of Cardio-Thoracic and Vascular Surgery, Skåne University Hospital, Malmö, Sweden

**Keywords:** Health service research, Meta-ethnography, Meta-synthesis, Qualitative studies, Older adults, Chronic low back pain

## Abstract

**Background:**

Of various chronic diseases, low back pain (LBP) is the most common and debilitating musculoskeletal condition among older adults aged 65 years or older. While more than 17 million older adults in the USA suffer from at least one episode of LBP annually, approximately six million of them experience chronic LBP that significantly affects their quality of life and physical function. Since many older adults with chronic LBP may also have comorbidities and are more sensitive to pain than younger counterparts, these older individuals may face unique age-related physical and psychosocial problems. While some qualitative research studies have investigated the life experiences of older adults with chronic LBP, no systematic review has integrated and synthesized the scientific knowledge regarding the influence of chronic LBP on the physical, psychological, and social aspects of lives in older adults. Without such information, it may result in unmet care needs and ineffective interventions for this vulnerable group. Therefore, the objective of this systematic review is to synthesize knowledge regarding older adults’ experiences of living with chronic LBP and the implications on their daily lives.

**Methods/design:**

Candidate publications will be sought from databases: PubMed, CINAHL, and PsycINFO. Qualitative research studies will be included if they are related to the experiences of older adults with chronic LBP. Two independent reviewers will screen the titles, abstracts, and full-text articles for eligibility. The reference lists of the included studies will be checked for additional relevant studies. Forward citation tracking will be conducted. Meta-ethnography will be chosen to synthesize the data from the included studies. Specifically, the second-order concepts that are deemed to be translatable by two independent reviewers will be included and synthesized to capture the core of the idiomatic translations (i.e., a translation focusing on salient categories of meaning rather than the literal translation of words or phrases).

**Discussion:**

This systematic review of qualitative evidence will enable researchers to identify potential unmet care needs, as well as to facilitate the development of effective, appropriate, person-centered health care interventions targeting this group of individuals.

**Systematic review registration:**

PROSPERO 2018: CRD42018091292

**Electronic supplementary material:**

The online version of this article (10.1186/s13643-018-0742-5) contains supplementary material, which is available to authorized users.

## Background

The average human life expectancy has increased significantly worldwide due to advances in medicine, health care delivery, and technologies over the recent years [1]. The The United Nations has estimated that the global population of people aged 60 or over will triple by 2050 [2]. The fast-growing aging population is accompanied by multiple health issues (e.g., musculoskeletal pain). It has been estimated that approximately 65 to 85% of older adults are suffering from musculoskeletal pain [3, 4].

Of various musculoskeletal pain, low back pain (LBP) is the most prevailing health condition in older adults that leads to functional limitations and disability [5–7]. LBP is defined as pain or discomfort between the 12th rib and above the gluteal sulcus with or without radiating leg pain [8]. More than 17 million older adults in the USA suffer from at least one episode of LBP annually [9]. Similarly, multiple population-based studies have found that the prevalence of LBP (regardless of chronicity) among community-dwelling older adults in the last 12 months ranged from 13 to 50% [3, 10–12]. Since the prevalence of chronic LBP increases with age [13–15], many older adults experience chronic LBP that lasts for at least 3 months [16, 17]. It has been estimated that prevalence rates of chronic LBP in individuals aged 60 years or older were approximately 30% in different parts of the world [12, 18]. In the USA alone, over six million older adults experienced chronic LBP that significantly compromised their quality of life and physical function. [9]. Importantly, since chronic LBP is the major contributor of disability (including falls [19]) in older adults [20, 21], its negative impacts extend beyond the patients. Chronic LBP (like other chronic pain) imposes severe financial burden to caregivers and society [22] although the direct impact of LBP on work productivity in retired older adults appears minimal.

Older adults with LBP face unique age-related vulnerabilities. Compared to younger individuals, older adults are more sensitive to pain because of the compromised endogenous pain modulation processing [23, 24] and decreased pain thresholds [25]. Additionally, comorbidities in older adults (e.g., cognitive impairment [26], polypharmacy [27], and multisource pain generation [28]) may potentiate the debilitating effects of chronic LBP [29], reduce patients’ adherence to medical and therapeutic interventions, and/or cause contraindications to LBP treatments [30]. Since older adults may also need to face multiple age-related psychosocial comorbidities (e.g., bereavement from the loss of spouse or friends, financial constraints, depression and social isolation [31–33]), these factors can negatively affect their LBP recovery, LBP-related disability, and attitudes/beliefs about pain [34].

Given the high prevalence of debilitating chronic LBP in older adults, designing and implementing proper age-related pain interventions has been suggested to be one of the priorities for educating healthcare professionals [35]. Although multidisciplinary chronic pain management approaches that incorporate the physician’s, nurse’s, and social worker’s perspectives have been recommended for treating older adults with chronic pain [36], patients’ perspective on their chronic LBP experiences is less emphasized in existing pain management guidelines [37]. Since chronic LBP can disrupt older adults’ life, as well as their family’s life and/or social relationships [36], it is paramount to look beyond medical treatments for these individuals so that more comprehensive approaches (e.g., the provision of supportive services or spouse participation) can be formulated to address age-related needs. For instance, a qualitative study interviewing a group of older adults with chronic pain living in rural Thailand revealed that these patients were more likely to adopt self-management programs when treatment for pain reduction or related information was more accessible, affordable, and acceptable [38]. Such information can inform the effective allocation of resources to meet patients’ needs.

While quantitative studies usually use theoretical-based self-reported questionnaires to evaluate the pain and function of older adults with chronic LBP [39], these studies are unable to determine the in-depth concerns or feeling of older adults with chronic LBP [40], which can explain patients’ behaviors and may better inform health and social policy for these patients. This limitation can be addressed by qualitative research. For instance, qualitative studies can provide insights into how age-related social roles can affect the older adults’ experiences with LBP. Therefore, a growing number of qualitative studies have been conducted to investigate the impacts of chronic LBP on various facets of life (e.g., coping strategies and social roles) in older adults residing in different settings [41–46]. However, no systematic review of qualitative studies has ever been conducted, and we propose to utilize qualitative evidence synthesis (QES) to integrate these research findings. Since age, gender, social class, levels of education, culture, and living environments may have differential influences on the perceived impacts of chronic LBP in older adults, QES can be applied to re-interpret the conceptual data from primary studies [47] in order to deepen the understanding of how chronic LBP impacts the life experience of older adults. Furthermore, QES can enrich the relevance of findings from multiple qualitative studies, thereby broadening the perspectives [48] and enabling to inform healthcare policy or practice [49].

Given the above, the overarching objective of this systematic review of qualitative research is to synthesize and conceptualize daily life experience of older adults living with chronic LBP. It will pose two specific questions to the included studies:

- What concepts concerning older adults’ experiences of daily life, when living with chronic LBP, can be identified?
- How can the identified concepts be understood (i.e., conceptually clarified)?

## Method/design

Meta-synthesis is often used as an overarching umbrella term for describing different methods of qualitative evidence synthesis. Meta-ethnography [50], the approach used in the current review, is the most commonly cited method in health service research to synthesize findings from studies with a qualitative design. Our rationale for conducting a meta-ethnography, instead than for example a thematic analysis, is that it is an interpretative rather than an aggregate form of knowledge synthesis. Accordingly, it aims to develop a conceptual understanding of the phenomenon [50].

### Search strategies

The search strategy, design, and structure for this meta-synthesis was prepared in accordance with the Preferred Reporting Items for Systematic Reviews and Meta-Analyses statement for protocols [51] (Additional file 1). A thorough search strategy will be developed using the SPICE (setting, population/perspective, intervention, composition, and evaluation) tool [52, 53], which will help formulate search terms for qualitative research studies with appropriate sample and phenomenon of interest (Table 1). An initial scoping search has been conducted on PubMed to understand how studies are indexed and what terms to use in searching titles and abstracts (Additional file 2). There was no date range limitation for the search. The initial search also aimed to check the suitability of the method of review and review questions and to estimate the number of expected citations for screening [54, 55]. The search supported the feasibility of formulating our tentative review questions, as well as the suitability of our method of synthesis (i.e., meta-ethnography).

**Table 1:** Overview SPICE

Setting	Perspective	Interest/intervention	Comparison	Evaluation
All settings and contexts	Older people 65+	Chronic low back pain	Not applicable	Daily living

Potential studies will be searched from PubMed (Additional file 3), PsycINFO, and CINAHL, acknowledged to cover the important quantity of health service research, by using keywords and their equivalent subject headings (e.g., Medical Subject Headings or CINAHL Subject Headings). Three search strings will be included: (chronic low* back pain OR chronic LBP OR CLBP OR chronic backache OR chronic non-specific low back pain OR chronic non-specific LBP OR lumbar pain OR lumbago) AND (qualitative study OR qualitative research OR action research OR ethnographic research OR grounded theory OR phenomenological research OR naturalistic inquiry OR focus group OR interview OR narrative) AND (older adults OR old people OR older persons OR older individuals OR geriatric OR seniors OR elderly). Adjacent syntax (older adj2 adult*) will also be used to identify papers involving older adults. A manual search of reference lists of the included studies will be performed. Forward citation tracking will be conducted using Scopus to identify additional relevant articles that were cited in the included studies.

### Selection of studies

Studies will be included if they (i) are in English, French, German, Spanish, Swedish, or Chinese; (ii) are published in peer-reviewed journals; (iii) used qualitative methods and a qualitative analysis; (iv) included participants aged 65 years and older regardless of study context; and (v) report on the experience of older adults with chronic nonspecific LBP. The latter is defined as pain in or near the lumbosacral spine with or without radiating leg pain that lasts for at least 3 months unrelated to osteoporosis, infection, tumor, fracture, cauda equina syndrome, and inflammatory disorders. There will be no limitations regarding the types of qualitative designs in the current meta-ethnography based on the suggestion from studies discussing the methodology of meta-ethnography [56, 57].

Studies will be excluded if (i) they used mixed methods where qualitative data cannot be extracted or (ii) the data analysis lacks the necessary conceptual depth (i.e., assessed as containing only non-translatable second-order concepts) [58]. A two-stage screening approach will be used. At the first stage, two independent reviewers (AW and GB) will screen titles and abstracts for the selection of articles for full-text screening. Any disagreement between the two reviewers will be resolved through discussion. If disagreement persists, the article under scrutiny will be included for full-text screening. At the second stage, all selected full-text articles will be retrieved. The same reviewers will use the previously applied selection criteria to perform full-text screening. Any disagreements will first be resolved through discussion, and if it persists, a third reviewer will make the decision for inclusion or exclusion of articles.

### Data extraction and data synthesis

This study will adopt an inclusive approach to extract data. Specifically, reviewers will extract all relevant data presented in a study using a standard form developed based on the suggestions from Toye et al. [58]. The extracted data will include study objectives, study design, authors, year of publication, context, sample size and demographics (gender, age, living circumstances), data collection methods, data analysis, and clearly articulated second-order constructs (e.g., concepts). Notably, second-order constructs, together with the text underpinning the construct, will be extracted. According to Schutz [59], a first-order construct is the phenomenon/experience perceived by the participants. A second-order construct is the researchers’ interpretation of the participants’ first-order constructs. In this review, all extracted concepts should explain the data (i.e., to be translatable). Two independent reviewers will exclude all second-order concepts that are deemed to be non-translatable or are irrelevant to the review questions. According to Toye and colleagues [58], this is a useful strategy for interpreting data of qualitative studies. Any disagreements between the two reviewers in regard to second-order concepts will be discussed with the research team, whereas a third reviewer will be consulted if disagreements among reviewers persist.

The extracted second-order constructs and their underpinning text will subsequently be transferred to a Word document (i.e., to a synthesis matrix). The latter will contain free space for the non-linear process of condensing the supporting content into idiomatic translations (i.e., translations focusing on salient categories of meaning rather than the literal translations of words or phrases [60]). According to Campbell and colleagues (2011), it is the translated data that gets synthesized so that a full understanding of the concepts can be reached [61]. The matrix will assist in identifying similarities and differences within and across included studies. Lines-of-argument (LOA) synthesis will be used to capture the core of the idiomatic translation. Specifically, the LOA synthesis will help interpret various translatable concepts within and across studies to construct the main core meaning (i.e., provide an overarching conceptual understanding) [60].

### Assessment of the methodological quality of included studies

In this review, methodological quality appraisal of the included studies will be conducted by the two independent reviewers because it is important to account for study quality when evaluating their overall influences on the end results. The appraisal is influenced by Toye et al.’s [58] conceptual model to quality appraisal, which suggested two core facets of quality for inclusion in meta-ethnography: (1) conceptual clarity (the clarity of the authors in articulating a concept that facilitates theoretical insight) and (2) interpretive rigor (what is the context of the interpretation; how inductive are the findings; has the interpretation been challenged?). The research team will develop an appraisal sheet in accordance with Toye et al.’s [58] conceptual model. The included studies will then be categorized as either a key paper, satisfactory paper, fatally flawed paper, or an irrelevant paper. If there are any disagreements between the two reviewers, a third reviewer will be consulted for decision-making. Our experience from previously conducted meta-ethnographies has shown that a study can be deemed as fatally flawed methodologically but it can still provide interesting and rich insights (Authors, under review). Therefore, we will include conceptually rich studies even when the methodological quality may be suboptimal. A sensitivity analysis of findings based on the quality of the included studies will be conducted [58].

## Discussion

Since the current systematic review aims to encompass relevant qualitative research papers related to experiences of older adults with chronic LBP, it will be a comprehensive benchmark review paper in the field. The findings not only will provide an overview of all relevant qualitative research areas but also will identify research gaps for future studies and can be used as a foundation for developing adequate assessments and/or interventions for older adults with chronic LBP. Specifically, this review will significantly contribute to the holistic management of chronic LBP in older adults by (1) integrating and synthesizing existing qualitative research findings related to chronic LBP experiences of older adults residing in different settings; (2) providing an in-depth knowledge with regard to older adults’ experiences of living with chronic LBP and the implications on daily life; and (3) identifying potential unmet care needs of this population so that more effective person-centered healthcare and social interventions can be developed/implemented. The findings emerging from the proposed project will subsequently form the theoretical and empirical basis for designing a research program to investigate the effectiveness, adequacy, and feasibility of culturally competent interprofessional allied health interventions in improving the health and independence of living in older people with chronic LBP.

## Additional files

Additional file 1: Prisma-P checklist. (DOCX 30 kb)

Additional file 2: Overview search filters PubMed. (DOCX 18 kb)

Additional file 3: Search string PubMed. (DOCX 16 kb)

## Abbreviations

LBP: Low back pain
SPICE: Setting, population or perspective, intervention, composition, and evaluation
