# Supplementary material for: Older adult’s experience of chronic low back pain and its implications on their daily life: Study protocol of a systematic review of qualitative research
Source: Syst Rev. 2018 May 24;7:81. doi: 10.1186/s13643-018-0742-5 (PMC5968538; doi:10.1186/s13643-018-0742-5)
Supplement: Supplementary file 2 — Overview search filters PubMed. (DOCX 18 kb) [file 13643_2018_742_MOESM2_ESM.docx]

Overview search filters PubMed

| **No** | **Search terms for block 1** (2017-11-09) | **No of hits** |
| --- | --- | --- |
|  | Aged [MeSH] | 2731141 |
|  | Geriatric assessment [MeSH] | 22764 |
|  | Retirement [MeSH] | 8684 |
|  | Aged [Title/Abstract] | 475793 |
|  | Elderly [Title/Abstract] | 213 730 |
|  | Frail*[ Title/Abstract] | 15 076 |
|  | Older* [Title/Abstract] | 349360 |
|  | Old age” [Title/Abstract] | 25 044 |
|  | Retire*[ Title/Abstract] | 17 348 |
|  | Senior* [Title/Abstract] | 33 461 |
|  | Geriatric* [Title/Abstract] | 54 887 |
| 12. | #**1OR#2OR#3OR#4OR#5OR#6#7OR#8OR#9OR#10OR#11** | **3,288,318** |

| **No** | **Search terms for block 2** (2017-11-09) | **No of hits** |
| --- | --- | --- |
| 13. | Low back pain [MeSH] | 18 276 |
| 14. | “Chronic LBP” [Title/Abstract] | 781 |
| 15. | CLBP [Title/Abstract] | 1165 |
| 16. | ”Chronic back pain” [Title/Abstract] | 1607 |
| 17. | ”Chronic backache” [Title/Abstract] | 86 |
| 18. | ”Chronic lumbar pain” [Title/Abstract] | 68 |
| 19. | ”Chronic lumbosacral pain” [Title/Abstract] | 10 |
| 20. | ”Chronic spinal pain” [Title/Abstract] | 161 |
| 21. | “Low back pain” [Title/Abstract] | 22659 |
| 22. | “Lower back pain” [Title/Abstract] | 1969 |
| 23. | Lumbago [Title/Abstract] | 1258 |
| 24. | **##13OR#14OR#15OR#16OR#17OR#18OR#19OR#20OR#21OR#22OR#23** | **32,359** |

| **No** | **Search terms for block 3** (2017-11-09) | **No of hits** |
| --- | --- | --- |
| 25. | Focus groups [MeSH] | 22 956 |
| 26. | Qualitative research [MeSH] | 34 726 |
| 27. | Nursing methodology research [MeSH] | 16 094 |
| 28. | Ethnogra* [Title/Abstract] | 8654 |
| 29. | Ethnolog* [Title/Abstract] | 1154 |
| 30. | Field study [Title/Abstract] | 6770 |
| 31. | Focus group* [Title/Abstract] | 34 412 |
| 32. | Grounded theory [Title/Abstract] | 9020 |
| 33. | Hermeneutic* [Title/Abstract] | 3069 |
| 34. | Narrativ* [Title/Abstract] | 27367 |
| 35. | Phenomenogra* [Title/Abstract] | 434 |
| 36. | Phenemenolog* [Title/Abstract] | 21 670 |
| 37. | Lived experience* [Title/Abstract] | 3992 |
| 38. | Life experience* [Title/Abstract] | 4070 |
| 39. | Qualitative [Title/Abstract] | 177035 |
| 40. | Experience* [Title/Abstract] | 891491 |
| 41. | Interview* [Title/Abstract] | 292929 |
| 42. | **#25OR#26OR#27OR#28OR#29OR#30OR#31OR#32OR#33**  **OR#34OR#35OR#36OR#37OR#38OR#39OR#40OR#41** | **1,283,443** |
| 43. | **#12 AND #24 AND #42** | **1,302** |
