# Supplementary material for: Older adult’s experience of chronic low back pain and its implications on their daily life: Study protocol of a systematic review of qualitative research
Source: Syst Rev. 2018 May 24;7:81. doi: 10.1186/s13643-018-0742-5 (PMC5968538; doi:10.1186/s13643-018-0742-5)
Supplement: Supplementary file 3 — Search string PubMed. (DOCX 16 kb) [file 13643_2018_742_MOESM3_ESM.docx]

Completed search string (#43) PubMed n= 1302 hits

((((((((((((((((((((("Focus Groups"[Mesh]) OR "Qualitative Research"[Mesh]) OR "Nursing Methodology Research"[Mesh]) OR ethnogra*[Title/Abstract]) OR ethnolog*[Title/Abstract]) OR field study[Title/Abstract]) OR focus group*[Title/Abstract]) OR grounded theory[Title/Abstract]) OR hermeneutic*[Title/Abstract]) OR narrativ*[Title/Abstract]) OR phenomenogra*[Title/Abstract]) OR phenomenolog*[Title/Abstract]) OR qualitative[Title/Abstract])) OR life experience*[Title/Abstract]) OR lived experience*[Title/Abstract])) OR experience*[Title/Abstract]) OR interview*[Title/Abstract])) AND ((((((((((("Aged"[Mesh]) OR "Geriatric Assessment"[Mesh]) OR "Retirement"[Mesh]) OR aged[Title/Abstract]) OR elderly[Title/Abstract]) OR frail*[Title/Abstract]) OR older*[Title/Abstract]) OR "old age"[Title/Abstract]) OR retire*[Title/Abstract]) OR senior*[Title/Abstract]) OR geriatric*[Title/Abstract])) AND (((((((((((lumbago[Title/Abstract]) OR "lower back pain"[Title/Abstract]) OR "low back pain"[Title/Abstract]) OR "chronic spinal pain"[Title/Abstract]) OR "chronic lumbosacral pain"[Title/Abstract]) OR "chronic lumbar pain"[Title/Abstract]) OR "chronic backache"[Title/Abstract]) OR "chronic back pain"[Title/Abstract]) OR CLBP[Title/Abstract]) OR "chronic LBP"[Title/Abstract]) OR "Low Back Pain"[Mesh])
